# Bibliometric structured review of tuberculosis in Nigeria

**DOI:** 10.4314/ahs.v23i2.16

**Published:** 2023-06

**Authors:** Sunday Adewale Olaleye, Oluwafemi Samson Balogun, Frank Adusei-Mensah

**Affiliations:** 1 School of Business, JAMK University of Applied Sciences, Rajakatu 35, 40100 Jyväskylä, Finland; 2 School of Computing, University Eastern Finland, Kuopio Campus, FI-70211, Finland; 3 Institute of Public Health and Clinical Nutrition, University of Eastern Finland, Kuopio, Finland

**Keywords:** Tuberculosis, bibliometric, University, Nigeria, collaboration, authors

## Abstract

**Background:** The tuberculosis burden is growing in Nigeria along with its population. For example, Nigeria has the sixth highest TB burden globally, with an estimated 4.3 per cent multi-drug resistance in new cases. This study builds on the existing study that examined academic involvement in tuberculosis research. The study in question focused on global medical literature related to tuberculosis, but the non-visibility of some low and middle-income countries in the bigger global picture motivated this present study. Every year, over 245,000 Nigerians succumb to tuberculosis (TB), with approximately 590,000 new cases reported (of these, around 140,000 are also HIV-positive). This study carried out an academic publication evaluation with the VOS viewer tool to map bibliometric data for scholarly articles published between 1991 and 2021 on tuberculosis research and used the Biblioshiny app for analytics and plots of authors, sources, and documents to explore the descriptive statistics of tuberculosis literature. The present study delineates that England has the highest collaborating country with Nigeria in the study of tuberculosis over the years and according to the report, the University of Nigeria, the University of Ibadan, and Nnamdi Azikwe University are Nigerian institutions with extensive collaborations. This study concludes with managerial implications for future actions.

## Introduction

The killer disease called tuberculosis is ravaging lives globally. However, its negative impact is more pronounced in the low and middle-income countries^1^, and the tuberculosis burden is one of the major concerns of the United Nations Sustainable Development Goals (SDGs) as the UN is working towards 90% tuberculosis deaths reduction by 2030^2^. The ambitious goals set by the UN emphasized the significance of tuberculosis research. TB infection occurs in four stages: the first is macrophage response, the growth stage, the immunological control stage, and the cavitation stage in the lungs. The initial macrophage reaction is the first step of the infection. These four steps take place over approximately one month.

## The epidemiology of tuberculosis

As one of the oldest known human pathogenic infectious diseases, the Mycobacterium tuberculosis (M. tuberculosis) complex tuberculosis is at a constant state of continual research globally^3^. The disease has a great economic and health impact globally. There has been a continuous effort to prevent and control TB with progress chalked in the development of therapeutic regimes. However, despite the long historical and continuous effort, *M. tuberculosis* infections remain a dogged health concern globally. It is of great concern due to the emergence of antibiotic-resistant MDR and XDR strains and the co-prevalence of diabetes and the HIV epidemics which increase TB risk^4^-^5^.

The evolving nature of the causative agent has resulted in the development of extrapulmonary TB and drug-resistant variants in addition to the respiratory burden of the disease. Currently, about 25% of the global population are either infected with active TB or have latent asymptomatic tuberculosis, with an annual incidence of 10 million new infections and 1.2 million mortality^3^. About 15% of tuberculosis cases occur in the form of extrapulmonary infections (infection affecting other parts of the body than the lungs). Such cases have been reported to be particularly difficult to diagnose and treat and are easily spread via organ transplant^6^.

A positive TB diagnosis using the routine TB test is by the measurement of TB immunoreactivity determined by either a tuberculin skin test (TST) (89%- 95% sensitivity)^7^, the QuantiFERON-TB Gold In-Tube test (QFT-GIT) (Cellestis Limited, Carnegie, Victoria, Australia) QFT-GIT (83% sensitivity) or by the interferon-gamma release assay (IGRA)^6^-^7^. The use of immunoreactivity as a proxy for infection is based on the principle of the body's natural immune response against the TB bacterial; that is, immunological memory and recall responses are used as markers for TB infection8. The immunocompromised have weakened immune responses and are prone to TB infection. Again, at low bacteria population in the immunocompromised, there is a possibility of false negativity using this testing approach. The body's cell-mediated immunity, especially the CD4+ T lymphocytes is a critical defence mechanism against the TB^6^. This observation justifies why HIV patients with weakened CD4+ defence mechanism HIV are at increased risk of TB infection. Active and future research on host tolerance and the mechanism of pathogenic elimination is warranted for better understanding. On the contrary, however, HIV patients have been shown to have lower sputum bacillary loads of *M. mycobacteria* and, therefore, are less TB infectious/contagious than the non-HIV patients^5^,^9^. Thus, low clearance of the pathogen from the system via the sputum.

## Pathogenesis

*M. tuberculosis* is a droplet airborne disease mainly caused by M. tuberculosis. Infection occurs when the tubercle bacilli droplet nuclei reach the alveoli of the lungs. The pathogen (tubercle bacilli) is engulfed by endocytosis of the alveoli's macrophages as a wall of defence against the pathogen. The pathogen spread via the lymphatic channels, the bloodstream, the lymph nodes, the apex of the lung, kidneys, brain, and bone in a case of immune barrier failure. This process of multiplying primes the immunological memory for a systemic response i.e. Latent Tuberculosis Infection (LTBI)^10^. The LTBI are detectable with the tuberculin skin test (TST) and interferon-gamma release assay (IGRA)^7^. The promotion of surveillance and continual use of these routine methods are of great interest.

The bacterial has the ability to form intracellular cords10. The mutation and cord forming ability of the pathogen has increased its virulence and resistance to drugs^10^-^11^. In the immunocompromised, the tubercle bacilli overcome the immune system and multiply, resulting in progression from LTBI to TB disease state. In general, the lifetime risk of progressing from LTBI or TB exposure to TB disease is about 10% and it increases with co-morbidities like diabetes and HIV. The lifetime risk increases by 30%/ among diabetic patients and 10% /year among HIV infected persons^5^.

Though TB most commonly affects the lungs; (pulmonary TB), it can affect other anatomical parts of the body including the lymph nodes, kidneys, brain, bones and joints and be transmissible via organ or blood transfusion (extra-pulmonary TB). In 2006, 18% of TB cases in the United States^12^ and 15–25% of all cases of TB were extra-pulmonary^13^.

**Figure 1:**
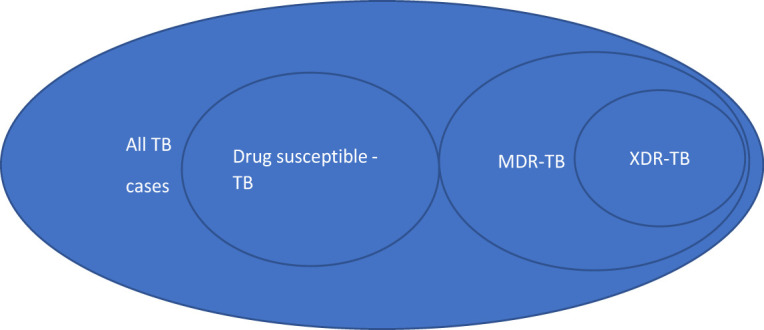
Multi-drug resistance TB. TB=tuberculosis, MDR=multidrug resistance and XDR= Extensively drug-resistant

Drug-resistant TB (primary/secondary) is caused by M. tuberculosis organisms that are resistant to first-line TB drugs. In multidrug-resistant TB (MDR TB), the organisms are resistant to the first-line TB drugs; isoniazid and rifampin^14^. In extensively drug-resistant TB (XDR TB), the organism is resistant to isoniazid and rifampin, and any of the second-line drugs (fluoroquinolone or the injectables; amikacin, kanamycin, or capreomycin)^15^ (See figure 1).

## TB Nomenclature System

For health care provision and surveillance purposes, TB stages have been classified into 5 distinct classes; class 0 is ‘no TB exposure (Not infected)’, class 3 is ‘TB clinically active’, class 4 is ‘Previous TB disease (not clinically active)’, and class 5 is ‘TB suspected (TB disease)^16^. Different active surveillance programmes are targeted to the class 3 and 5 TB groups for identification and treatment. The current study builds on the existing study of Nafade et al.^17^ that examined academic involvement in tuberculosis research for almost a decade (nine years). The study in question focused on global medical literature that is related to tuberculosis. The Non-visibility of some low and middle-income countries in the bigger global picture motivates the current study. Every year, over 245,000 Nigerians succumb to tuberculosis (TB), with approximately 590,000 new cases reported (of these, around 140,000 are also HIV-positive). In Nigeria, tuberculosis (TB) is responsible for more than 10% of all deaths. Every hour, approximately 30 individuals die because of the condition, even though there are effective therapies available^18^. The cited statistics reflect around 181.1 million in 2015 of the Nigerian population, but six years later, Nigeria's population has increased to 213,173,116 as of November 19, 2021, which indicates a 17.8% increase.

The tuberculosis burden is growing in Nigeria along with its population. For example, Nigeria has the sixth highest TB burden globally, with an estimated 4.3 per cent multi-drug resistance in new cases in the country. Another study later shows that Nigeria moved from sixth to seventh position worldwide and became the second in Africa comparatively with thirty countries highly burdened with tuberculosis^19^-^20^. However, the country had one of the lowest case detection rates in the world in 2018, with an estimated 24 per cent of incident cases being detected - far lower than the WHO STOP TB objective of 84 per cent21. As a result of the lengthy treatment period, which can last several months, and the resulting loss of earnings, tuberculosis diagnosis is not always straightforward in Nigerian hospitals and clinics. Because of Nigeria's large population, increasing detection rates, increasing primary health care capacity, and detection of drug-resistant infections all necessitate a significant investment of financial resources. In this situation, a delay in treating a tuberculosis patient can potentially result in the transfer of the infections. The earlier disruption of tuberculosis intervention is HIV, but the ongoing COVID-19 has also compounded the challenge of tuberculosis attack. Some victims of tuberculosis also have HIV in addition to coronavirus.

Tuberculosis is creating more global attention, and it is a research domain that is worth investigating. Some recent studies expanded the literature on tuberculosis with focused on the South-Eastern part of Nigeria and shed more light on health system factors that affected the implementation of the tuberculosis control program with concentration on the service providers and discovered that lack of human resources management is a bottleneck of tuberculosis service delivery. The authors also discovered that prevalence and development of tuberculosis drug resistance in the community were both influenced by factors such as combination therapy, multiple therapies, and non-compliance with treatment^22^-^23^, evaluation of the prevalence of drug-resistant tuberculosis and focus on trend and determinants of tuberculosis treatment^20^.

Further, another study examined the challenges Nurses at a Federal Teaching Hospital in Nigeria faced while caring for tuberculosis patients^19^. The advanced research in the South-Eastern is like that of Southern-Western of Nigeria, and Oladimeji et al.^24^, Akande^25^ evaluated the trend and predictors of successful tuberculosis between 2010 and 2016 and evaluated the levels of TBIC-related knowledge and behaviours among nurses in Ibadan, Oyo State, Nigeria, and the socio-demographic factors that influence these levels. In North Central Nigeria, a study examined the smear-negative pulmonary tuberculosis in a high HIV burden patient1, while another study focused on Southern Nigeria and investigated the unmet needs of patients undergoing multidrug-resistant tuberculosis treatment^26^. These scientific papers portray tuberculosis as a national burden.

All these papers contributed to the tuberculosis literature, but bibliometric analysis on tuberculosis is scarce in Nigeria. This study fills the gap of bibliometric contribution to the tuberculosis literature and answers the following research questions: (1) What is the milestone and impact of scientific production of tuberculosis in a developing country setting? (2) How did collaboration impact the scientific work of tuberculosis in a developing country setting and the segmentation of the co-authorship? (3) What are the emerging trends of author's keywords and author's keywords plus? The second section of this study detailed the methodology employed, while the third section captured the results. The fourth section concluded the study with study limitations and future direction of tuberculosis.

## Methodology

This study followed the six steps of systematic bibliographic workflow utilized in Olaleye's^27^ study, as showcased in Figure 1. This study designed three unique research questions in step one and used network analysis - bibliographic coupling, co-word, co-author, and co-citation to answer the unanswered research questions. In step two, the study found the Web of Science more appropriate for data sources to search, filter, and export bibliographic data. In step three, the present study carried out bibliometric data analysis VOSviewer and Bibiloshipny App. In step four, the current study used VOSviewer and Biblioshiny App to visualize the tuberculosis data. In Step Five, this study presents the results generated from VOSviewer and Biblioshiny and makes necessary interpretations based on the results. Finally, in step six, the study concludes with a summary of the findings, expresses the study's limitations, and proposes future studies.

Bibliometrics denotes statistical methods to analyze academic publications and get insights into the body of knowledge for a specific or combined subject of inquiry. The bibliometric technique is based on examining bibliographic data from published literature^28^. The bibliometric method is used in this work to attempt to understand the phenomenon of tuberculosis. When looking for relevant literature, the researchers used the following search query to find their results: It is possible to find ((Abstract-Title-Keyword (“tuberculosis”)) in a top global database (ISI Web of Knowledge database) and excluded the languages French, Spanish, German, and Russian from your search. Studies done in the English language were included.

**Figure 2:**
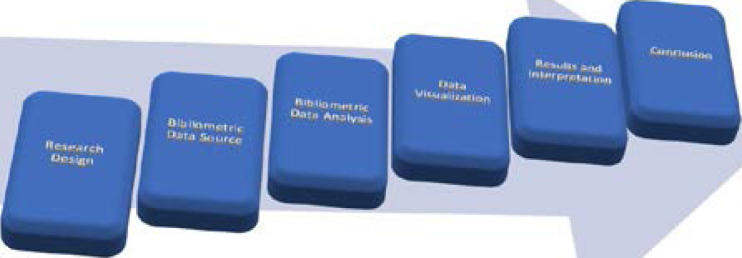
Bibliographic Systematic Workflow

When it comes to the bibliometric approach, this study draws inspiration from Olaleye^27^, and Figure 2 depicts an updated methodology that has been put into practice. The Web of Science database, a high-quality collection of academic intellectual documents, was used to research and assess pertinent literature. Researchers used the phrase “Tuberculosis” to search for academics from 1975 to 2022. They found 164,434 academics between 1975 and 2022. The study was limited to three decades (1991-2021), English, and articles due to the focus on tuberculosis. As a result, the number of academic works on tuberculosis was reduced to 755. The study used 50 papers for the analysis as suggested by the Web of Science Core Collection algorithms. The refinement criteria and search terms are as follows.

**Table:** 

Topics include “tuberculosis” (Topic) and Articles (Document Types) and NIGERIA (Countries/Regions) and 1991 or 1992 or 1993 or 1994 or 1995 or 1996 or 1997 or 1998 or 1999 or 2000 or 2001 or 2002 or 2003 or 2004 or 2005-2021 (Publication Years) and Social Sciences Citation Index (SSCI) or Emerging Sources Citation Index (ESCI) or Science Citation Index (SCI) and Social Sciences Citation Index (SSCI) (Web of Science Index).

This study carried out an academic publication evaluation with the VOSviewer tool^29^ to map bibliometric data for scholarly articles published between 1991 and 2021 in tuberculosis research. It is a quantitative technique that provides an analytic framework for studying the structure of tuberculosis literature as well as the interactions between items with various attributes, keywords, co-citations, and coupling maps, among other things. The mapping and network visualization techniques are used to investigate the tuberculosis literature, with clusters and empty gaps indicated throughout the investigation. As Aria and Cuccurullo^30^ recommended, the study used the Biblioshiny app for analytics and plots of authors, sources, and documents to explore the descriptive statistics of the tuberculosis literature with data illustration.

## Results and discussion

### Data Synthesis

The dataset's summary information is presented in Table 1. Using the table below as an example, how many different sorts of documents were collected can be seen. The article type has the most documents (n=47). An article: proceedings paper (n=2) follows, followed by an article; early access (n=1). As used in this study, the author's keywords relate to a precise list of keywords that authors of a publication have provided (typically less than ten) to characterize what their study centred on as used in the full text. Keyword plus refers to expanded keywords and phrases created by the Web of Science algorithm that includes keywords from references mentioned by authors of a publication^31^. Additionally, authors per document refer to the mean number of authors per document, and co-author per document refers to the average number of authors' appearances per document. Both authors per document and co-author per document are used to assess the level of collaboration between authors.

**Table 1:** Tuberculosis bibliometric descriptive

Description	Results
Timespan	1991:2021
Sources (Journals, Books, etc)	35
Documents	50
Average years from publication	7.55
Average citations per document	7.3
Average citations per year per doc	0.9065
References	1
**Document Types**	
article	47
article; early access	1
article; proceedings paper	2
**Document Contents**	
Keywords Plus (ID)	135
Author's Keywords (DE)	136
**Authors**	
Authors	242
Author Appearances	267
Authors of single-authored documents	1
Authors of multi-authored documents	241
**Authors Collaboration**	
Single-authored documents	1
Documents per Author	0.207
Authors per Document	4.84
Co-Authors per Documents	5.34
Collaboration Index	4.92

**Figure 3:**
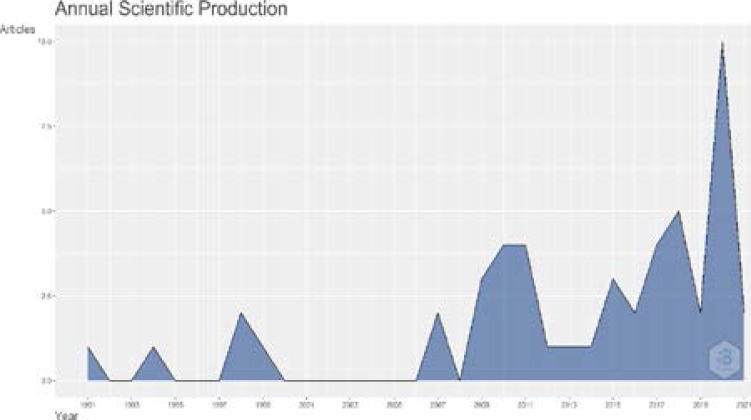
Annual Scientific Production

**Figure 4:**
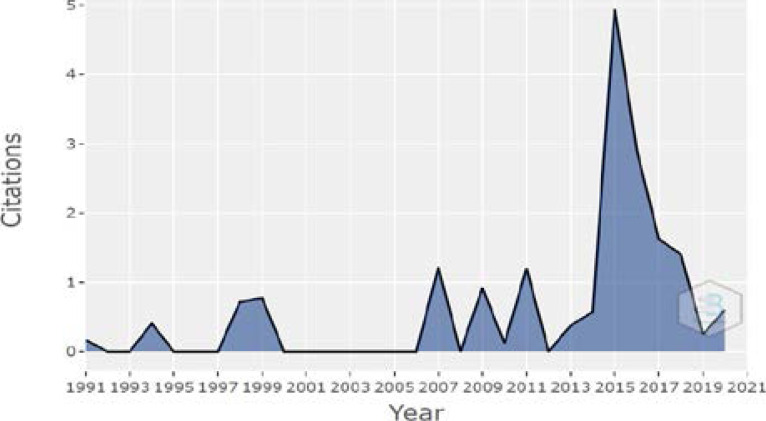
Authors Citation

From 1991 until 2021, this graph depicts the yearly production of papers about tuberculosis (see figure 3). According to results generated from the bibliometrix R package, the study of tuberculosis has a 4.16 per cent yearly growth rate in scientific output from 1991 to mid-2021. Four papers were reported in 2010, 2012, and 2017, indicating the start of the study's spectacular development in publishing. In 2018, 5 papers were published, resulting in a significant increase in the number of publications published. In 2020, ten papers were published, marking the largest number of publications published in a single year to date. Because the research of tuberculosis settings is still in its early stages, the scientific contribution is likely to expand year after year, as demonstrated by the results of the analysis (figures 3 and 4).

Figure 4 depicts the number of citations received each year from 1991 to 2021. Citations show a cyclical pattern from 1991 to 2021. Even though tuberculosis is still a deadly illness in many African nations. Between 2015 and 2016, there is a significant increase in citations, which then decreased in 2017. Furthermore, even though the year 2020 has the most publications published, the citation rate is low.

The study investigated the social structure component of the tuberculosis literature with bibliometrix R-package regarding the co-authorship and social cooperation analysis30, that is available in the biblioshiny user interface (UI). Scholars define a social network of players within a field as the interaction between two or more individuals, institutions, or nations in terms of partnerships^32^-^33^.

**Figure 5:**
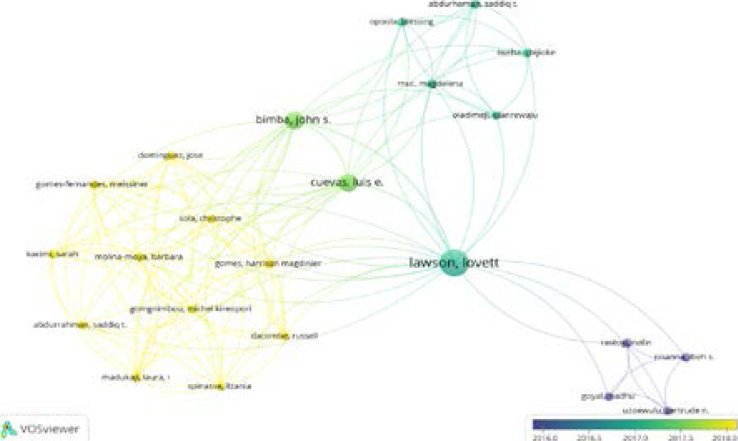
Co-authorship

These relationships are shown in the form of a network, with nodes representing actors and connectors linking the nodes representing relationships. The collaboration network between authors is depicted in Figure 5. The findings indicate that the large names already listed as productive researchers in the discipline, such as Bimba, John, Cuevas, Luis, Lawson, and Lovett. These authors have a well-established collaborative network.

**Figure 6:**
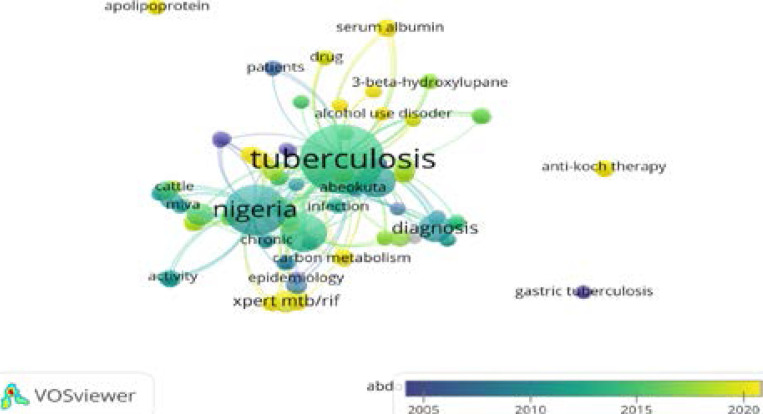
Authors Keywords

Figure 6 displays the grouping of terms used by writers in their scientific publications. Authors frequently use keywords such as tuberculosis, cattle, mlva, infection, carbon metabolism, chronic, activity, epidemiology, xpert mtb/ rif, alcohol, 3-beta hydroxylupane, usage, disorder, patients, drug, serum albumin, TB, Nigeria, and diagnostic. The graph illustrates that three keywords stood out as being commonly utilized by authors. Words like TB, Nigeria, and diagnostics. The image also highlighted terms that were not commonly used by writers, such as gastric tuberculosis, anti-Koch treatment, and apolipoprotein.

**Figure 7:**
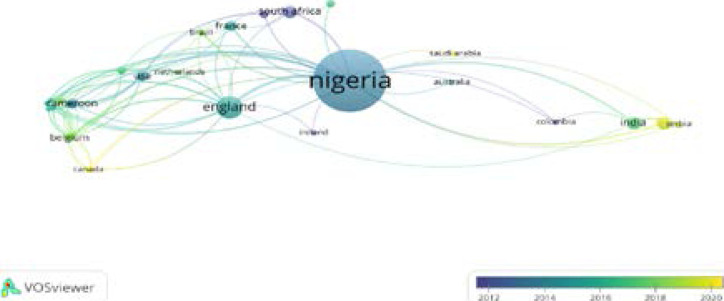
Collaborating Countries

Nigeria plays a central role in Tuberculosis research collaboration. The countries on the left and right sides of the big node of Nigeria are the nations that collaborate with Nigeria on themes linked to tuberculosis, as reflected in Figure 7. Country representatives from Africa, Europe, South America, and Asia are among those who engage with Nigeria.

**Figure 8:**
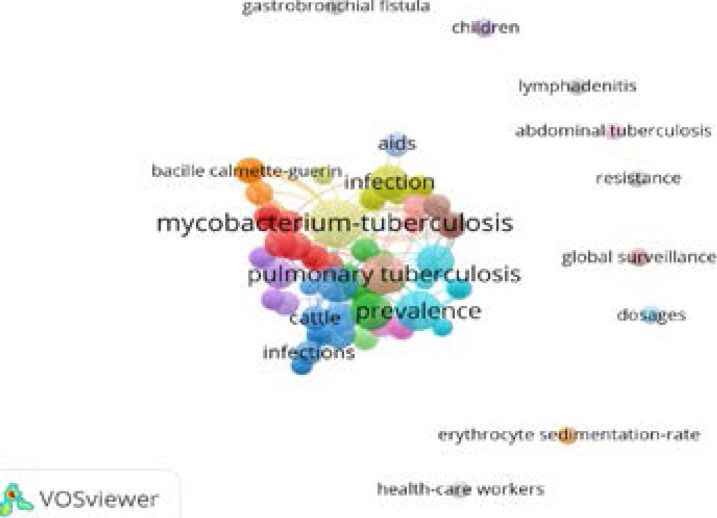
Authors Keywords Plus

This graph depicts the terms that are often used in TB research. These are terminologies that are only used in the study of TB. Words like mycobacterium-tuberculosis, the bacteria that causes tuberculosis, pulmonary tuberculosis, TB, cattle, Bacille Calmette-Guerin, infection, and AIDS. Furthermore, the chart includes terms that are not usually used in TB research, such as doses, resistance, abdominal tuberculosis, global resistance, health-care workers, and erythrocyte sedimentation-rare (see figure 8).

**Figure 9:**
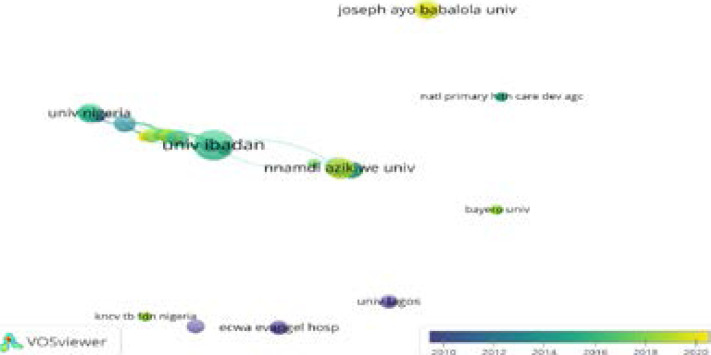
Organizations

Figure 9 shows some institutions such as the University of Nigeria, the University of Ibadan and the Nnamdi Azikwe University all in Nigeria are seen to have created a big network of collaborations among themselves. However, a few other universities are shown to have little or no collaboration network. Although these institutions are actively contributing to the research in the study of Tuberculosis, they have not established collaborations with other institutions to expand their social network in the field. For example, Joseph Ayo Babalola University KNCV TB FDN Nigeria, ECWA Evangelical Hospital, Bayero University Kano, National Primary Health Care Development Agency, and the University of Lagos are in isolation with no collaboration network.

Document's citation assessment is one of the key parameters in research quality assessment. The author performance has been ranked based on citations. In the case of an equal number of citations, the one with the higher linked strength is given preeminence. Based on citations, the top three authors are Abimbola Seye, Aghanwa, HS and Agbaji, o. with (74, 32 and 12) citations respectively. Abimbola, seye's paper received as twice the citations as compared to the Aghanwa, HS in the second position which indicates the high impact of Abimbola's paper on Web of Science in the last 30 years. Abimbola's paper represents an average citation of 2.46/ year which is over 250% of the average citations per year per document (0.9889/year/document) for the 50 extracted papers. However, with regards to linked strength, the papers of Adetunji, Adegboye, Adejumo, Adejumo et al. and Adesola ranked top based on the linked strength (10, 9, 9, 9 and 9) respectively. From the data, an average of 4.34 co-authors per document were observed in the present study (meta table, not shared).

Again, 243 core authors have published at least 1 paper on tuberculosis, and no author has published more than one paper accounting for the productivity of 4.86 core authors per document (n=50) or 0.21 authors per document which indicate that the topic area in the field of medicine is in the stage of rapid development^34^.

It is imperative to note that, research flourishes well in an atmosphere of collaborative research and such an assessment is important in establishing further future collaborations for upcoming researchers in the field or strengthening the existing research collaborations. Trust and confidence are paramount in building collaborations^35^. Document IDs linked to multiple institutions / institutional IDs indicates the collaborative effort of different institutions in producing such a document (s). In table 3, it could be observed that most documents were produced by multiple institutions in collaboration for advancing knowledge in tuberculosis research. For instance, documents with IDs 6, 7, 8 and 9 were each produced by at least 3 institutions (table 3) while ids 11-18 were each produced by at least a collaboration of 4 different institutions. Transdisciplinary collaborative research has been proved to be of great potential in speeding up the adoption rate of evidence-based practices in health care^36^. The unique challenge that tuberculosis presents in terms of multidrug resistance development, identification and longevity in therapeutic prognosis serves as an excellent opportunity, provide incentives and presents collaborative environments for research professionals of diverse skillsets and institutions to collaborate to forge progress in this field of research^37^.

**Table 3:** Organizational relationship

Document id	Organization id	Frequency
1	2	1
2	87	1
3	64	1
4	94	1
5	57	1
5	104	1
6	3	1
6	18	1
6	107	1
7	1	1
7	63	1
7	94	1
8	22	1
8	34	1
8	72	1
8	89	1
9	11	1
9	64	1
9	65	1
10	53	1
10	93	1
11	21	1
11	27	1
11	33	1
11	76	1
11	95	1
12	8	1
12	16	1
12	90	1
12	92	1
12	110	1
13	64	1
13	96	1
14	33	1
14	36	1
15	24	1
15	43	1
15	91	1
16	15	1
16	23	1
16	25	1
16	69	1
16	74	1
16	94	1
17	1	1
17	23	1
17	29	1
17	45	1
17	63	1
17	102	1
17	109	1
18	19	1
18	81	1
18	82	1
18	85	1
19	21	1
19	33	1
19	76	1
20	6	1
20	73	1
20	77	1

The assessment of tuberculosis research institutions clarifies the core institutions in this research domain. There are in total 109 research institutions and the top-3 of them produced 6 out of the 50 papers accounting for 6% (n=50). From the top 20 institutions, dga mris mission rech & innovative sci is the most influential institution with 29 citations and a linked strength of 4 (table 4), while brown univ and ctr muraz & natl tb program both has a total of 22 citations and a linked strength of 22 each occupying the second position. Institutions with the most productive authors were observed to be ahmadu bello univ, bingham univ and abia state univ with each having a total of 2 documents each from Web of Science on tuberculosis. It is interesting to note that, the number of documents does not always correlate with the number of citations and the linked strength as could be observed in the present study, a trend that was also observed in a previous study34. The institutions with the greatest number of documents had 9, 4 and 4 citations respectively for ahmadu bello univ, bingham univ and abia state univ compared to the highest citation of 29. In addition, a total of 0, 11 and 8 linked strengths respectively were recorded for ahmadu bello univ, bingham univ and abia state univ compared to the highest linked strength of 22 (table 4).

**Table 4:** Organization

id	organization	Documents.	Citations.	total link strength
1	dga mris mission rech & innovat sci	1	29	4
2	brown univ	1	22	22
3	ctr muraz & natl tb program	1	22	22
4	ahmadu bello univ	2	9	0
5	bayero univ	1	6	2
6	ahmadu bello univ zaria directorate anim health & livestock	1	5	2
7	dev	1	5	2
8	bingham univ	2	4	11
9	Abia state univ	2	4	8
10	dept publ health	1	4	4
11	covenant univ	1	4	0
12	delta state ministry health	1	2	5
13	antwerp univ	1	1	10
14	bernhard nocht inst trop med ctr global health security &	1	1	10
15	diplomacy	1	1	10
16	ambrose alli univ	1	1	2
17	bobo dioulasso	1	0	7
18	douala tb reference lab chukwuemeka odumegwu	1	0	3
19	ojukwu univ	1	0	2
20	dr dv patil univ	1	0	2

**Figure 10:**
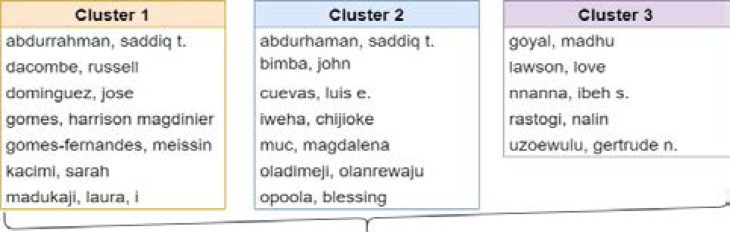
Authors names

Evaluating authors' performance and currently, active researchers in the field are key to knowing the key authors in that field of research^34^. Based on the top 20 authors, the authors in cluster 1 forms the most prolific class of authors in tuberculosis research in Nigeria with the most recent publications in the field based on the number of citations, documents published and the linked strength in the past 3 years (since 2018) (figure 5). In cluster 2 of figure 10, active authors with documents published in the past 4 years (since 2017) are presented whiles cluster 3 represent authors with publications in tuberculosis published in WoS in the past 5 years (the year 2016) (figure 5, 10).

**Figure 11:**
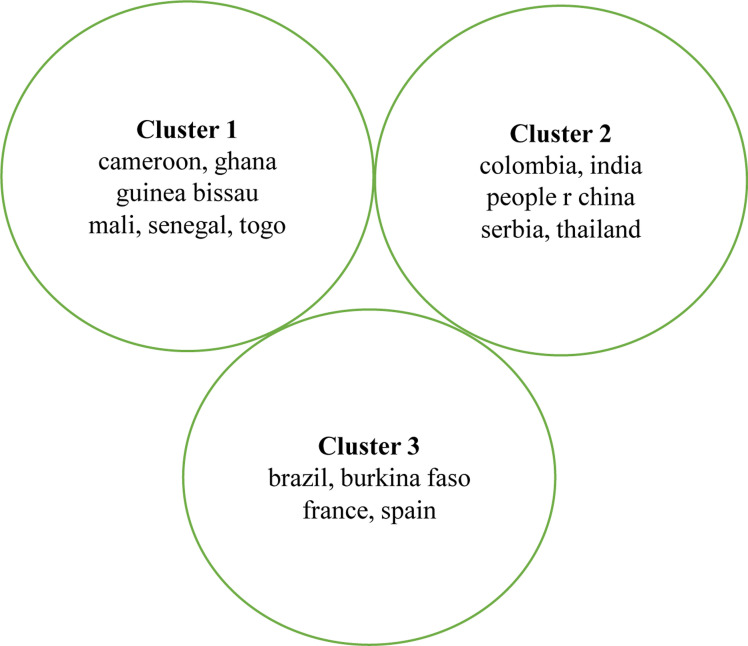
Authors country

Built on the bibliographic data from WoS, the countries for authors and co-authorship network visualization map are created by VOSviewer (figure 6); the minimum document threshold of a country is 5 and there are 15 countries out of 29 listed items. Nigeria, Cameroon, Ghana, Guinea Bissau, Mali, Senegal, and Togo are identified as the cluster 1 countries with the largest number of studies due to the location of the leading groups of researchers and practitioners in tuberculosis situated in the above countries or areas. However, Nigeria as a country has the greatest contribution to the publication and impact in tuberculosis research carried out in Nigeria (figure 6). Most of the tuberculosis research in the country is led by active local researchers within the four corners of the country. However, the shortlist of over 28 other countries indicates the strong network between the local researchers and researchers affiliated with foreign institutions (figure 7, 11). With Nigerian tuberculosis research as a mirage of the global issue, the countries in the three clusters typify the global interest of tuberculosis research in developing countries like Nigeria and in Africa as a whole where over 25% of the TB deaths occurs^38^. Africa (cluster 1), Europe, (France, Serbia and Spain), cluster 3, South America (Colombia) and Asia (India and Thailand) 2 shows the global interest in TB research in Nigeria.

**Figure 12:**
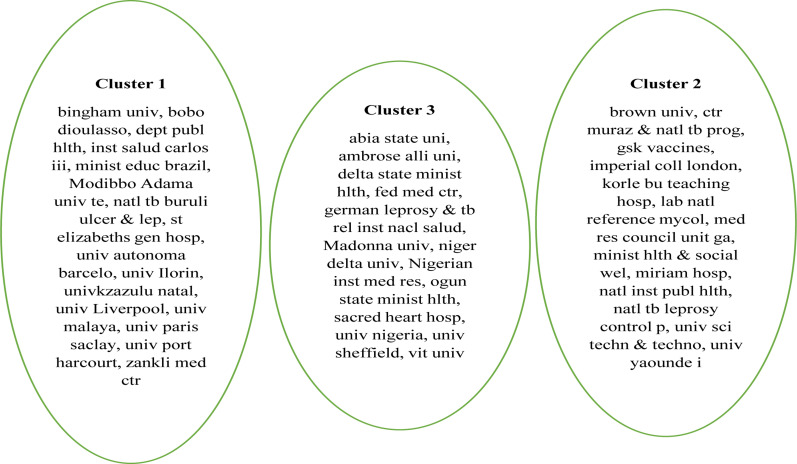
Authors Institution

The analysis of tuberculosis research institutions can clarify the distribution of the core institutions in this area of research^34^. The authors' institution and their network with other leading institutions are presented. Bingham University in cluster 1 is a private and one of the few universities with more than 1 publication on TB and located in Karu, 25 near Abuja is networked with other leading universities in the field including univ of Liverpool in the UK. Brown University in Providence, Rhode Island of the United States of America, and Abia State in Uturu town, Nigeria in clusters 2 and 3 respectively shows the wide distribution of both local and international universities in TB research in Nigeria (figure 12). Again, the active involvement and collaboration of foreign institutions including Brown University in the USA and Liverpool University in the UK in TB research to some extent justify the founding pattern awarded by foreign institutions towards the TB research in Nigeria with over 217 funds awarded by USA based institutions alone during the study window (table 5). The clusters represent the global and geographical distribution with the University of Ibadan, Nnamdi Azikiwe University, and the University of Nigeria as some of the major local players in the field. These three universities churn out close to 50% of the tuberculosis research papers in the country (figure 9).

**Table 5:** Funding Agencies

SN.	Agencies	Output
1	United States Department of Health Human Service	64
2	National Institutes of Health NIH USA	52
3	European Commission	41
4	United States Department of Health Human Service	36
5	Medical Research Council UK Mrc	29
6	Nih Fogarty International Center Fic	23
7	United States Agency for International Development	22
8	Bill Melinda Gates Foundation	17
9	Welcome Trust	17
10	Cigar	16
11	World Health Organization	16
12	Nih National Institute of Allergy Infectious Disease	15
13	National Institute for Health Research Nihr	12
14	Centres for Disease Control Prevention USA	10
15	Nih National Institute of Mental Health NIMH	10
16	Nih national Institute on Agung Nia	10

The fundamental authors in the research community have important link strength to promote the development of the tuberculosis research discipline. The authors are connected in many regards to the co-authors, institutions and countries influencing the linked strength with clusters as shown above. As such, they form an important part of the bibliometric analysis^39^-^40^.

The table shows the funding agencies sponsoring TB research. With United States Department of Health Human Service leading with 64 and closely followed by the National Institutes of Health NIH USA with 52 and then the European Commission with 41 and the least funding agencies are Centers for Disease Control Prevention USA, Nih National Institute of Mental Health Nimh and Nih national Institute on Agung Nia with frequencies of 10. The agencies made it easy for researchers to come up with innovative research in the study of TB as funds are released to worthy research. The funding distribution indicates the importance of Tuberculosis research.

The discovery of the involvement of body's cell-mediated immunity, especially the CD4+ T lymphocytes and the host's susceptivity to the disease is a milestone in TB research^6^. Such observation throws more light on why HIV patients with weakened CD4+ defence mechanism HIV are at increased risk of TB infection. The mechanism behind this observation is still far from reach warranting active and future research on host tolerance and the mechanism of pathogenic elimination for better understanding. The complex nature of the disease, the special adaptation of the pathogen including the recently discovered intracellular cord forming ability^10^ demands collaborative research from researchers and authors from diverse background with different skillset and geographical exposure to make impact^13^. The identification of the collaborative networks of different researchers in the current paper is a plus towards TB research.

## Conclusion

With the use of bibliometric analysis, this research attempted to give a comprehensive evaluation of scholarly publications in the field of tuberculosis over many decades. The study investigated the themes of tuberculosis in the publications; it recognized prolific scholars and their contributions; it explored social networks and collaborations across institutions, countries, and regions over time; and it presented the thematic analysis of the study of Tuberculosis by showing its status in terms of the themes, as well as its prospects for the future in terms of those themes.

Similarly, our result delineates that England has the highest collaborating country with Nigeria in the study of tuberculosis over the years. According to the report, the University of Nigeria, the University of Ibadan, and Nnamdi Azikwe University are all Nigerian institutions with a large network of collaborations. In terms of authors contributing to the study of TB, the following authors received the most citations: Abimbola, Seye, Aghanwa, HS, and Agbaji, O., who received (74, 32, and 12) citations, respectively. In addition, the research found that TB research has lately evolved to include new and rising features including mycobacterium-tuberculosis, the bacteria that causes tuberculosis, pulmonary tuberculosis, TB, cattle, baccile calmete-guerin, infection, and aids. Further, this study answered the three unanswered research questions in the literature in the context of a developing country. One, this study reveals the milestone and the impact of scientific production of tuberculosis in Nigeria based on the three decades of literature on tuberculosis by indigenous and foreign authors. Two, this study shows densely and disjointed collaboration by home and foreign authors and the need for the enhancement of the segmentation of the co-authorship. Three, the author's keywords shows the necessity of extensive tuberculosis diagnosis in Nigeria while the keywords plus emphasized the prevalence of different types of tuberculosis. This study suggests that the tuberculosis managers in Nigeria should pay attention to the prevailing existing and emergence of different types of tuberculosis in Nigeria and seek intervention to curb the burden. The managers should find more innovative early diagnoses of tuberculosis in Nigeria and appeal to human resistance to treatment. Furthermore, tuberculosis should strategies to reduce human error among the medical practitioners that specialize in tuberculosis. For the academic community, there is a need for more local and foreign collaboration to advance tuberculosis research. Finally, the continual mutation of the pathogen, increased emergence of MDR and XDR and the identification of increased susceptibility of HIV and diabetic patients to TB calls for increased collaboration and funds allocation towards understanding the mechanism behind these processes.

## Limitation and future study

This study was limited to Nigeria as one of the epic centres of tuberculosis in Africa. Also, there are some other drawbacks to this study. To a large extent, the study's weaknesses are related to the sampling of data. The study was unable to merge data from multiple databases because of a technological constraint in the software utilized to analyze. This analysis relied on a sample drawn from the Web of Science database, which may have omitted important information. The study would be vastly improved if it drew data from numerous independent databases. Though we use inclusion and exclusion criteria judiciously, but it is not out of place that some papers miss out. To expand the scope of this study the Search terms should also be adjusted to include more relevant keywords when searching the database. In the future, researchers should investigate ways to collect data from numerous databases with broader keywords to conduct a more inclusive study. The study's findings are intended to inform academics, mainly young scholars in innovative learning contexts, about the current state of research and potential future hotspots. For example, this study will help the future researchers to identify hot papers, productive authors, and funding agencies that counts in the TB research domain. There are some new themes in the TB study that need to be further explored to link the study's objectives. The findings of this study give a quick summary of the production in this field over the years and a meaningful hint to the future direction of TB research. This paper highlights some future research proposals in the study of TB.

(1) It may be necessary to build more comprehensive research collaborations between academics and institutions, so generating a greater worldwide influence on TB research to find strategies to combat the disease.

(2) It is proposed that scientists devote more time and effort to learning analytics, machine learning, and deep learning since the study indicates that these are future research issues relevant to TB.

## Figures and Tables

**Table 2:** Author performance

No.	author.	documents.	Citations.	total link strength
1	Abimbola Seye	1	74	2
2	Aghanwa Hs	1	32	1
3	Agbaji O.	1	12	3
4	Adegboye Oluwatosin	1	10	9
5	Adejumo Olusola adedeji	1	10	9
6	Adepoju Victor	1	10	9
7	Adesola Sunday	1	10	9
8	Agbakoba N. R.	1	9	7
9	Adewole I. F.	1	7	6
10	Abubakar I.	1	6	5
11	Adamu A. l.	1	6	5
12	Afieroho O. E.	1	6	4
13	Abdulkadir Alhaji idris	1	5	5
14	Abdulmalik Zainab	1	5	5
15	Adetunji M. A	1	4	10
16	Abdurhaman Saddiq T.	1	4	7
17	Adewole, Olufemi o.	1	3	3
18	Adedokun Kamoru	1	2	3
19	Aghaji M. N.	1	1	1
20	Abdurrahman Saddiq T.	1	0	3
